# Perceptions of stigma among people with lived experience of methamphetamine use within the hospital setting: qualitative point-in-time interviews and thematic analyses of experiences

**DOI:** 10.3389/fpubh.2024.1279477

**Published:** 2024-02-13

**Authors:** Cheryl Forchuk, Jonathan Serrato, Leanne Scott

**Affiliations:** ^1^Lawson Health Research Institute, London, ON, Canada; ^2^Arthur Labatt Family School of Nursing, Western University, London, ON, Canada

**Keywords:** qualitative research, substance use, health equity, primary healthcare, education and training

## Abstract

**Objectives:**

As part of a larger mixed-methods study into harm reduction in the hospital setting and people with lived experience of methamphetamine use, stigma was found to be a prominent issue. The aim of this secondary analysis was to investigate the issue of stigma.

**Design:**

Participants completed a one-time qualitative interview component to assess their experiences in the hospital setting.

**Setting:**

The study setting included secondary and tertiary care in Southwestern Ontario, Canada. Participants who had received care from these settings were also recruited from an overdose prevention site, a primary healthcare center, a national mental health organization, an affordable housing agency, and six homeless-serving agencies between October 2020 and April 2021.

**Participants:**

A total of 104 individuals completed the qualitative component of a mixed-methods interview. Sixty-seven participants identified as male, thirty-six identified as female, and one identified as non-binary. Inclusion criteria included past or current use of methamphetamine, having received services from a hospital, and being able to communicate in English.

**Methods:**

Open-ended questions regarding experiences in the hospital setting were asked in relation to the lived experience of methamphetamine. A secondary analysis was conducted *post-hoc* using a thematic ethnographic approach due to prominent perceptions of stigma.

**Results:**

Three themes were identified. The first theme identified that substance use was perceived as a moral and personal choice; the second theme pertained to social stigmas such as income, housing and substance use, and consequences such as being shunned or feeling less worthy than the general patient population; and the third theme highlighted health consequences such as inadequate treatment or pain management.

**Conclusion:**

This study revealed that stigma can have consequences that extend beyond the therapeutic relationship and into the healthcare of the individual. Additional training and education for healthcare providers represents a key intervention to ensure care is non-stigmatizing and patient-centered, as well as changing hospital culture.

## Introduction

Amphetamines and prescription stimulants are the third most used class of substances worldwide, with North America having the highest rates of use (1). A recent study from British Columbia, Canada, found a rise in self-reported rates of methamphetamine use, from 59.7% (*n* = 290) of participants in 2018 to 71.7% (*n* = 445) in 2019 (2). This corresponds with a national increase in methamphetamine loads found in wastewater analysis (3) and an increased rate of reported stimulant-related harms, largely associated with methamphetamine use (4). Increasing rates of methamphetamine use may be linked to several factors, including its widespread availability, low cost, and increasing purity (5). In British Columbia, the presence of methamphetamine in drug toxicity-related deaths increased from 20.7% in 2012 to 43% in 2022 (6), and methamphetamine was involved in 52% of apparent stimulant toxicity deaths across Canada between January and September 2022 (7). In Ontario, there was an increase of more than 200% in population-based emergency department visits related to amphetamines between 2015 and 2020 (8).

Among individuals who concurrently use methamphetamine and opioids, methamphetamine has been reportedly used as a means to offset withdrawal and sedating side effects of opioids and opioid antagonist therapy, elicit a certain high/experience (9, 10), and support the ability to complete activities of daily living, self-protection, and survival (9–11). Methamphetamine may also be viewed as a safer alternative to other substances and may just be a preferred substance of use (10).

The concept of stigma can be defined as holding negative and prejudicial attitudes toward an individual, demonstrating behaviors such as humiliation, patronizing, providing insufficient information, disregarding an individual’s capacity for responsible action, and reinforcing cultural stereotypes (12). Three types of stigmas that may be experienced by people who use substances include self-stigma (the internalization of negative messaging surrounding substance use resulting in low self-esteem and feelings of shame), social stigma (negative attitudes, behaviors, labels, and images directed toward people who use substances or their friends and family), and structural stigma (barriers created due to health and social service policies) (13).

Perceived attitudes of healthcare professionals may play a critical role in perpetuating the stigma and discrimination faced by people who use substances (14). A systematic review by van Boekel et al. (15) examining articles published between 2000 and 2011 noted healthcare professionals typically held stigmatizing attitudes toward people who use substances. The stigmatizing attitudes of healthcare professionals toward people who use substances can manifest in many ways, such as victim blaming or shaming, the use of labeling language (e.g., “junkie”), the perception that they do not care about their own health, perceptions of drug-seeking behavior, the withholding or provision of substandard care (16, 17), and pain mismanagement (18–20).

Experiences of stigma by people who use substances can result in barriers to care and adverse health outcomes such as missing scheduled medical appointments (21, 22), avoiding medical care and diagnostic testing (23, 24), delaying seeking medical care (25), prompting people who use substances to leave care settings against medical advice (18, 26), and deterring stigmatized individuals from disclosing substance use to healthcare professionals (16, 22, 23, 27).

The widespread stigmatization faced by people who use substances in healthcare settings creates a need for focused interventions that address stigma on every level (14). Hospital-based harm reduction strategies may support a reduction in substance-related stigma of patients and staff, resulting in enhanced feelings of wellbeing among people who use substances (28). Although there may be a vast amount of literature on the stigmatization of people who use substances, there is limited literature on the stigmas associated with methamphetamine use (29). The present study is part of a larger mixed-methods project into harm reduction strategies to be implemented in hospital settings for methamphetamine use. The primary aim of the overall study was to explore current experiences, needs, and attitudes pertaining to harm reduction strategies and hospital care for people with lived experience of methamphetamine use. One of the study’s research questions specifically pertained to the experiences of the hospital setting among people with lived experience of methamphetamine use. A prominent concern was the perception of stigma from healthcare professionals toward people with lived experience of methamphetamine use, thus prompting a secondary analysis to further understand the issue. The purpose of this article is to investigate the qualitative findings related to stigma and methamphetamine use and to understand the potential consequences of such findings for people who use methamphetamine.

## Method

### Design

The overall study utilized a purposive sampling design by aiming to enroll a diverse range of participants based on age group, sex, ethnicity, and types of services accessed (e.g., hospitals, homeless-serving agencies, an affordable housing agency, primary healthcare agencies, mental health organizations, and an overdose prevention site) in Southwestern Ontario. Hospital settings included secondary and tertiary care. The rationale for targeting people accessing a variety of services was due to the difficulty of accessing people who use methamphetamine in hospitals. Those identifying as a minority (e.g., sexual minority/LGBTQIA2S+ [lesbian, gay, bisexual, transsexual, queer, intersex, asexual, 2-spirited, plus], Indigenous, and ethnic minority) were prioritized to ensure sufficient representation. The purpose of these interviews was to document the hospital care experiences of people who use methamphetamine and to gain insight into the changes necessary to implement harm reduction strategies in these settings. Ethical approval for this study was obtained from Lawson Health Research Institute and the Western University Research Ethics Board.

The focus of this article is to analyze the secondary findings pertaining to perceptions of stigma. The study adopted a mixed-methods design consisting of qualitative as well as quantitative interview components focusing on physical and mental health, service utilization, quality of life, substance usage, and community integration. The study utilized a participatory action research (PAR) approach that consisted of patient and public involvement. PAR can be defined as a method whereby participants as well as various stakeholders can influence and guide the research process with application to qualitative and quantitative study designs (30).

The study appointed patient and public representatives to the project’s advisory group, who provided input into the design, conduct, reporting, and dissemination plans in accordance with PAR. The advisory group was created at the start of the project and informed all aspects of the study, including recruitment, the development of research questions as well as questions for secondary analyses, and dissemination. Members of the advisory group consisted of researchers, physicians, nurses, nurse educators, senior hospital leadership, ethicists, overdose prevention site staff, social workers, homelessness agency representatives, a police department representative, and people with lived experience of methamphetamine use. The group, along with all its representatives, will inform the design of future interventions and policy changes. Interpretation of the findings was also discussed with the group, as were suggestions for further inquiry and secondary analyses. Dissemination of the results will be offered at a public event where all participants will be invited. Physical copies of the findings will be distributed by community partners as well as via outreach by the research team to all participants.

The interviews also allowed participants to become a part of the research design by informing them of interventions, harm reduction strategies, resources, and potential changes to practice, as well as directions for further research investigation.

### Recruitment

In order to be considered for the study, individuals must (a) have identified past or current experience using methamphetamine at any point in time; (b) be between the ages of 16 and 85; (c) have been admitted to the hospital for any reason during their methamphetamine use; and (d) have sufficient English language skills to complete an interview. Researchers reached out to the services/agencies participating in the project to ensure a diverse range of clients were represented. Among inpatients, referrals were also made by physicians and other healthcare providers who spoke to patients in their care. All potential participants were given a copy of the Letter of Information (consent form) and the research protocol which provided further details of the study. Hospital and agency staff spoke to interested individuals and explained the study in order to ascertain whether individuals would be interested in being contacted and be able to participate. Potential participants were also screened by hospital and agency staff prior to the interview to ensure they were able to provide consent. Research staff also spoke to potential participants to assess whether they were cogent to participate. Posters advertising study information were also displayed in community partner organizations and patient areas in hospitals. Recruitment began in October 2020 and concluded in April 2021.

### Procedure

After providing informed consent, participants took part in a mixed-methods interview. Interviews were conducted via telephone or in person. The interview consisted of qualitative, open-ended questions followed by a series of quantitative surveys focusing on demographics, substance use, health, quality of life, and service usage. Those that were in-person were conducted using appropriate personal protective equipment and physical distancing measures to abide by COVID-19 restrictions. In-person interviews occurred on-site at community agency locations and/or outdoors through outreach activities and pre-arranged interview sessions with agencies. Interviews performed via telephone required the researcher to read the consent form verbatim and allow the participant to stop for any questions or repeat specific sections as and when needed. A researcher-specific consent form was also completed, which documented the informed verbal consent provided. The mixed-method interview then commenced over the telephone with a voice recorder placed next to the telephone speaker for the qualitative component.

The qualitative portion of the interview was semi-structured and recorded using an audio recorder. The interview guide (see Appendix A) asked participants about their experiences in the hospital in relation to their lived experience of methamphetamine and harm reduction, including what they perceive to be current ongoing issues and what needs to be changed and not changed. Any question could be declined for an answer, and participants were given the option to leave at any time. Upon completion of the interview, participants were given a $20 honorarium as compensation for their time, either in-person or via e-transfer for telephone interviews, even if some questions were declined or the interview ended prior to full completion.

### Data analysis

Interview recordings were transcribed verbatim and then validated by another research team member to ensure the integrity of the transcript as well as de-identification. Transcripts were then analyzed using a thematic ethnographic approach, which seeks to develop a deep understanding of meanings and experiences within a social group (31). Interview transcripts were reviewed line-by-line to identify statements pertaining to stigma and how these impacted the individual. The transcripts were reviewed for similar words, phrases, and descriptors of stigma, which were then categorized into overarching, major themes. Quotes were color-coded based on the type of consequences experienced and then transferred from transcripts into a new master document to develop and explore experiences within each theme. Major themes were then developed as patterns and consequences of stigma began to emerge. No qualitative data software was used; the data were observed and transferred directly by the co-authors. A secondary analysis was then conducted on this major theme of stigma to identify the specific issues and consequences and how they potentially connected or influenced each other. Subthemes were then identified within each major theme and explored further, which allowed for a greater understanding of the contextual issues and what the consequences of these themes and subthemes resulted in.

To ensure the credibility and trustworthiness of the analyses, the themes identified and the subsequent model developed were conducted collaboratively and with consensus between the three authors, one of whom was the principal investigator. All three authors had considerable experience working with substance use and homelessness as frontline staff as well as researchers and were therefore well suited to addressing the research question. All three authors, as well as seven trained research assistants, performed the interviews. The authors were proficient in qualitative analysis with backgrounds in psychiatric nursing, psychology, and nursing, respectively.

## Findings

One hundred and four participants completed the qualitative component of the study. Interviews lasted approximately 1 h in length. A total of 67 participants identified as male, 36 identified as female, and 1 identified as non-binary (see Table 1). Thirteen participants identified as a sexual minority/LGBTQIA2S+, with one participant reporting they were “Questioning/Unsure.” The participants’ mean age was 35.5 years old. Furthermore, the majority of participants identified as Caucasian (*n* = 61) and single (*n* = 75). Of the participants who identified as Indigenous (*n* = 24), Indigenous and Caucasian (*n* = 8), or those categorized as other mixed race (*n* = 3), the following Indigenous groups were represented: Oneida (*n* = 6), Ojibwe (*n* = 3), Inuit (*n* = 3), Metis, Chippewa, Mi’kmaq, Six Nations, First Nation (all *n* = 2), Sisika, Mohawk, Algonquin, Anishinaabe, Cree, Ojibwe and Oneida, and U.S. Native American (all *n* = 1). One participant identified as Aboriginal, and four did not know their Indigenous background.

**Table 1:** Participant demographics.

	No. of participants (%)
**Mean age (SD), years**	35.5 (12.5)
Age range	17–66
**Sex**	
Male	67 (64.4)
Female	36 (34.6)
Non-binary	1 (1.0)
**Identified as a sexual minority/LGBTQIA2S+**	13 (12.5)
**Ethnicity**	
Caucasian	61 (58.7)
Indigenous	24 (23.1)
Indigenous + Caucasian	8 (7.7)
Black	3 (2.9)
Latin American	2 (1.9)
Other mixed race	6 (5.8)
**Marital status**	
Single	75 (72.1)
Married/common law/engaged	16 (15.4)
Separated/divorced	11 (10.6)
Widowed	2 (1.9)
**Highest level of education completed**	
High school	45 (43.3)
Grade school	41 (39.4)
College/university	18 (17.3)
**Current housing status**	
Homeless	52 (50.0)
Live alone	25 (24.0)
Inpatient (long-term admission)	7 (6.7)
Live with spouse/partner	6 (5.8)
Live with unrelated person	6 (5.8)
Live with other relative	5 (4.8)
Live with parents	3 (2.9)

Recruitment sites for the study included homeless-serving agencies (*n* = 27), an overdose prevention site (*n* = 22), a youth-serving agency (*n* = 22), a hospital (*n* = 18), low-income housing programs (*n* = 8), a primary healthcare center (*n* = 4), via an Assertive Community Treatment (ACT) team (*n* = 1), a community addiction support center (*n* = 1), and a community mental health crisis stabilization program (*n* = 1). Almost the entire sample reported experiencing homelessness in their lifetime (*n* = 102), of which 52 participants reported they were currently homeless or using a shelter.

A total of 70 participants had last used methamphetamine within the week prior to their interview, 45 of whom stated they used methamphetamine daily. No participants were removed from the study or required a rescheduled interview due to being under the influence. Although all participants had reported a physical (*n* = 87) or psychiatric hospitalization (*n* = 59) in their lifetime, 50 participants reported at least one physical hospitalization in the year prior to the interview, while 18 participants were admitted for a psychiatric hospitalization in the same timeframe.

### Thematic analyses

**Figure 1:**
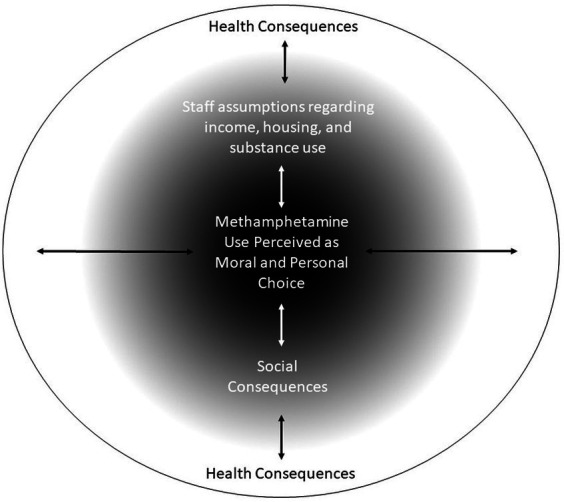
Conceptual model of stigma and methamphetamine use in relation to healthcare.

Stigma was identified by the majority of research participants as a major concern. A number of different consequences of stigma were identified by the participants, and a model was developed by the co-authors (see Figure 1). It is important to note that the three layers identified are not mutually exclusive but rather can influence and interact with one another. At the core of these stigmas was the notion that substance use was perceived as a moral and personal choice. In lay terms, this represents the belief that people with addictions have made “poor choices,” which they perceive has been asserted by healthcare professionals. This lack of understanding of addiction as an illness allows for the development of various stigmas, misunderstandings, and perceived feelings of judgment.

Around these perceptions of choice held by participants were two issues of stigma that were interlinked with each other: assumptions regarding income, housing, substance use, and social consequences. These two themes were largely formed based on experiences of negative patient–provider interactions. Moreover, these themes represented the intersectionality of the population and their interpersonal experiences within the hospital setting. Social consequences pertained to interactions and socialization with healthcare professionals, which included issues such as being ignored, shunned, and feeling dehumanized. Largely, these were based on the appearance of the participant and the immediate response or actions of the healthcare provider.

Patients may experience negative consequences in terms of their healthcare as a result. Health consequences include inattention to medical symptoms, inadequate care, and a lack of follow-up or help-seeking for future health conditions/complications. This may also have had an effect on discharge against medical advice as a result of health needs not receiving attention, such as inadequate withdrawal and pain management. This in turn could result in health concerns not being addressed or treated, readmission to the hospital, and/or further consequences for these particular individuals at a later date.

However, outside of these consequences, there was a minority of participants who reported positive experiences. These responses suggest that stigmatization is not prevalent across the entire healthcare system and that there are staff who are cognizant of the needs of people who use methamphetamine.

### Themes

#### Perceived as a moral and personal choice

There were recollections articulated by approximately a third of participants regarding healthcare providers suggesting their ill health was self-inflicted due to substance use and that to remedy their situation, the individual should just cease their usage. This was also associated with moral judgments of treatment in that presenting symptoms were considered to be the result of poor choices. Participants spoke about their perceptions of being less of a priority compared to other patient populations. It was also suggested that intravenous use was regarded as a more negative method of use compared to other ways of using substances.

Well I’ve been, you know, looked down at, because I’m an IV (intravenous) user and so a lot of times you feel like you’ve got a sign on you and that hospital staff is all like sort of pointing a finger, going “oh yeah” you know. It’s like they don't want to help you. Even if you have a legit problem, they make you feel like, “too bad you made this yourself.” (Participant 085)

Perceived adverse experiences among healthcare providers with people who use substances may influence interactions with future patients more negatively or regard them as “bad.” Participants discussed how they felt judged and that their addiction was not regarded as an illness.

Like maybe it could be almost like a good role model to show the staff at the hospital look just because they do drugs doesn't mean they're bad people. Yes, some of them are bad people, but they're not all bad people. You know what I mean? That’s what I hate. Like when like somebody treats me, like a piece of s**t, because they had an experience with somebody who did the same drug as I do and that person was, was garbage to them. So because of that, one experience alone, they treat everybody else that they know, that is like that. (Participant 094)

#### Staff assumptions regarding income, housing, and substance use

Approximately a quarter of participants believed that there was often stigma based on their housing or income status. Living in a shelter was perceived to be detrimental and indicative of intravenous substance use and/or resulted in perceptions of being treated differently from other patients. It was also highlighted that individuals who were perceived to be of lower socioeconomic status were also held in lower regard.

As soon as they find out you’re from down in this area, if you stay at the Men’s Mission or Salvation Army (homeless shelters), you’re automatically tagged as an intravenous drug user, and you’re treated like you’re less than. (Participant 058)

Participants discussed the concept of having their appearance judged based on specific features, including tattoos, teeth, and physical build. An unwell appearance may have been perceived as the result of poor choices and therefore is met with a different interaction compared to a patient who is healthier or is considered to be more presentable.

…which is weird because when, when I do get healthy and I, I usually get pretty muscular. I get big, I get healthy and you wouldn't be able to tell the difference. So anyway, when that does happen I'm totally, I’m treated totally different. (Participant 109)

#### Social consequences

The majority of participants reported many social-based stigmas, such as being seen as “just a drug addict or junkie,” with an emphasis on substance use as a negative behavior. There was a perception of being regarded as less than equal or different from the typical patient population. The experience of having concerns disregarded can then result in a poor therapeutic relationship.

Well, my experiences, it's just that like, when I go to the hospital, sometimes I feel like, you know, because of my methamphetamine use that people don't really listen to what I have to say or like I don't get the best of care. Like I feel like sometimes, like I'm being, they're kind of being biased the way they treat me … Like when, when I have concerns or something like, it just, I feel a little bit different, like I'm treated differently because of it, because I've been through mental health, like, I feel different than when I used to. (Participant 037)

It was also reported that, in addition to the stigma of being a “junkie,” there was also a lack of knowledge and understanding regarding methamphetamine use. These two combined can lead to ineffectual care being provided, reinforcing the perception that people who use methamphetamine are not seen as equal to other patients.

As I said before just like how people, like the people there, like they just like automatically assume that you, even if you didn’t do drugs, they just automatically assume. Like you come in, you’ve like been doing drugs, or you’re doing drugs or, and it’s just, they just like see you as just a person that’s not wanting to get off of the drugs or whatever … I think it needs a little bit more improvement. I find that like when I do go to the hospital they see you as like not, not everybody. They see me as, I’m just a junkie to them ‘cause like I do, I do drugs. (Participant 057)

Others discussed the feeling of being regarded as “a nobody” as well as perceptions of dehumanization. These descriptions were represented as a more hostile perception of healthcare professionals’ interactions and treatments and a significant imbalance of power between patient and practitioner. There were feelings of dehumanization, which was also perceived to lead to a lack of empathy as well as apathy toward providing treatment.

Yeah, being seen and you don’t feel, you know, they exist like they’re just dirt and they get treated like that it’s not, it’s not a good feeling. You’re not treated as human. Well I find that the hospital is very against it (harm reduction) and uncaring as soon as they find out, their attitude changes and you don’t get help that you deserve. (Participant 032)

As a result of this perceived underlying stigma, participants discussed how they felt like they were shunned or ignored by healthcare providers due to their substance use. Many reported informing healthcare providers of their substance use and felt perceptions of judgment and a lack of desire to provide them with healthcare.

It's saying like, like people they just need help more like, you know, like if, if a meth head comes to you and asks for help you should probably help them instead of judging them and the hospitals turn away so many meth heads. You know, before I got sober, I was turned away by five hospitals. (Participant 091)

Participants reported that they felt they were receiving unfair treatment compared to patients who do not use substances. It was after these perceived experiences that participants reported that they began to avoid using the hospital for care due to the discomfort of interacting with healthcare providers.

It makes me not want to reach out and ask for help from any medical people. Like because you’re pretty much shunned. Like my experience you know I was pretty much shunned everywhere that I went when it comes to like, like, whether it be, doctors, or, like any business type people, like, you know, they look down on you because you're a user, well, we're still human beings, right? (Participant 109)

#### Health consequences

A crucial outcome of all these themes was that patients who use methamphetamine reported that they believed they did not get treatment for the ailment that they sought medical attention for. It was suggested by approximately a third of participants that health issues were dismissed as opposed to a medical condition requiring attention, with pain relief an unmet need and mental illness mistaken for usage symptomology. Symptoms and reasons for being admitted were perceived to have been overlooked, with the individuals’ substance use taking precedence.

So like I've been shunned from the hospital just for being a drug addict. So when I went there for mental health issues. Because I was a drug addict and they just, they say all my problems were drugs. It wasn't like, because I suffer from PTSD and everything else. (Participant 105)

There were also perceived judgments around patients assumed to be demonstrating drug-seeking behavior. A lack of understanding as to how methamphetamine affects the individual, as well as withdrawal, may also influence this notion. It was also highlighted that symptoms may be overlooked, treated as a secondary concern, or regarded as an attempt to acquire medications. Care for the presenting symptoms may be lacking, less than satisfactory, or even ignored.

Like they don't necessarily give us the same care they would give somebody that, um, isn't a user. Like they just, I’ve had friends where they’ve experienced, they just assume that we're drug seeking, but there's actually something wrong, but they don't, they don’t take it seriously. (Participant 027)

People who use methamphetamine may not feel comfortable attending follow-up appointments, seeking further support, or receiving after-care following medical procedures. This could then have negative health consequences for the individual, who may need additional treatment and/or medical conditions that are left untreated due to self-stigmatization following negative experiences in the hospital.

Same with opiates. I've had an opiate problem, but if I go and sprain my knee, that I've had surgery on and my knee is this big and I can't move it, it's not fair that I don't feel comfortable going to the hospital to get an MRI, to see if the surgery worked. If it, if the surgery needs to be done again, I don't feel comfortable going because I feel already the premise is already there that they think I'm coming for pills. (Participant 089)

#### Positive experiences

Despite the large number of participants who reported negative perceptions pertaining to stigma, some participants did report that they received adequate care and support. The knowledgeability of some staff was praised, and there were healthcare providers who understood the perspective of people who use methamphetamine. This suggests that beliefs about stigmatizing behavior were not persistent in all interactions with healthcare providers.

She's the one that everybody asks for when they go to (name of hospital). She'll give you the opiates, if you need them she helps you. Doctor, oh, Doctor (name). I think she's a fantastic doctor. She really supports people that are users. You know, she makes sure that you, you know, get what you need when you're in there … You know, like the doctors were more understanding, like, you know, they were like, “I'm not here to judge you, we just need to, you know, know what your use is so that we can address the problem better than not knowing.” (Participant 085)

Positive interactions with nursing staff were also reported by a small number of participants, indicating that there are differences in attitudes toward people who use methamphetamine and that appropriate care is provided.

Whereas the third morning was the (name of different hospital) Hospital and there was nothing but “would you like some more fluids? Sleep some more, you need to catch up on sleep. So sleep as much as you can. You know, take your time. Is there anything else we can do for you?” … You're treated like an equal, you're treated with dignity. They have good bedside manner which falls in line with being professional, and non-judgmental and compassionate. (Participant 065)

## Discussion

Stigma regarding methamphetamine use and substance use in general was reported by almost all participants. This study not only echoed previous research studies but also revealed consequences specific to people who use methamphetamine. Specifically, this study found patients perceived their medical symptoms to be overlooked or dismissed as a result of symptom use. Mental health issues and being under the influence were reported to be conflated, which led to inadequate care being provided. This study also proposes a conceptual model that explains the core themes of stigma in this population such as moral choice, leading to negative social interactions resulting in health consequences.

The findings of this study reflect a previous study into substance use-related stigma in that two modes of stigmatization were largely experienced: judgmentalism and inattention (32). Such perceptions of judgmental biases and stigma may be founded on the idea of punitive prohibition, a collectivist philosophy in which community norms and values should shape individual freedom, with those in breach being held accountable (33). This would be closely linked to the theme of *Methamphetamine Use Perceived as a Moral and Personal Choice* with those choosing to break away from accepted societal norms and values are in some way acting immorally or contradictory. This in turn could create the foundation for further stigma and negative interactions, as reported in the study’s findings. In conjunction with this notion, stigma may be based on current supply/demand-reduction policies and legislation to eliminate use (34, 35). Current policies are aimed at reducing use, and so those in breach may be seen as making poor choices that healthcare providers must now address and repair through the provision of treatment. Policies emphasizing abstinence may have also predisposed participants to interpret interactions with healthcare providers as stigmatizing when rules and guidelines were enforced. It is possible that healthcare providers were merely maintaining and following the expectations laid out in policy and practice which may have been misunderstood as discriminatory on the individual level.

The study revealed that people who use methamphetamine experienced *Social Consequences* through perceptions of isolation, shunning, and judgment leading to an unwillingness to interact with the healthcare system in the future and self-isolation. Previous qualitative studies conducted in Canada have reported that people who use substances can become alienated within the healthcare setting due to the expectation of maintaining societal norms, which may be difficult when under the influence of substances (36). However, it may be that people who use substances and healthcare providers have different perceptions of stigmatizing behavior, resulting in misinterpretations and healthcare providers unintentionally acting in stigmatizing ways, despite their best intentions (37). The findings revealed that there were some positive interactions with staff, which indicates that stigma is not a consistent issue across all healthcare staff. There was an interplay between this theme and the theme of *Health Consequences* as participants claimed there was inadequate healthcare as a result. Symptoms may go overlooked as substance use becomes the primary focus, while others who do disclose usage may not receive pain medication due to concerns about drug-seeking behavior. It was reported that mental health crisis and substance symptomology overlapped, which can further exacerbate patient frustration.

Additional education for healthcare staff could represent a key implication of the study findings in order to facilitate de-stigmatization. It has been identified that enhanced education is needed for frontline healthcare providers caring for people who use methamphetamines, specifically management and treatment options (38). In collaboration with members of the advisory group, it was identified that there was a need for an education module to be enhanced to better support people who use methamphetamine in the hospital. A multi-component intervention in Canada for healthcare providers that incorporated educational workshops (including some with people with lived experience of substance use) was found to significantly reduce stigma and change attitudes toward mental illness, addiction, and recovery (39). In the present study, it was highlighted by a number of participants that they felt staff needed further training in how to interact with people who use substances. This is pertinent given that previous research has reported that individuals with less knowledge about methamphetamine are significantly more likely to hold stigmatizing attitudes (29). The findings of this study also suggested that further knowledge and education may be required in regard to substance use and withdrawal symptomology and issues such as drug-seeking behavior. Future research should aim to test the current knowledge of healthcare providers more definitively to pinpoint the gaps for further education.

There was a particular stigma toward intravenous use of methamphetamine, which has been previously reported in the literature (40). A study into methamphetamine use and harm reduction services in British Columbia, Canada reported that predominant attention to intravenous practices may have inadvertently created a gap in knowledge and a lack of direction in response to harms that may arise from smoking substances (41). Therefore, education should focus on enhancing healthcare providers’ knowledge of methamphetamine use (38), incorporating people with lived experience in education (39), and raising awareness about stigmas (42), which, in turn, could reduce the stigmatization faced by people who use methamphetamine in hospital settings.

A key strength of this study was collecting qualitative data from 104 people with lived experience, which exceeded the typical sample size of saturation, and a range of collective experiences and perspectives contributed to this analysis. These findings provide an overview of the current hospital experience rather than reporting on singular or individual issues. Furthermore, this study aimed to recruit a diverse sample of individuals, and although the number of female and sexual minority/LGBTQIA2S+ participants was lower than anticipated, there may have been fewer had the study not focused on underrepresented populations. Forty-one percent of the sample identified as an ethnic minority, which was greater than expected and offered representation that may not have been possible without the purposive sampling strategy.

A potential limitation of these findings is that the study focused specifically on people who use methamphetamine. Although participants who reported polysubstance use were eligible, this may have led to other experiences and perspectives being missed. Another limitation that was identified pertained to participants being interviewed predominantly from one medium-sized city in Canada. Other locations with larger or smaller populations may report greater or lesser experiences of stigma as well as different consequences of perceived discrimination. Differing ethnocultural issues may also have an influence on potential future findings, particularly as the sample for the current study was predominantly Caucasian. Future research needs to be conducted in different locations in order to assess the scope of stigma experienced by people who use substances. Research focusing specifically on the experiences of other ethnic minorities would provide vital and unique qualitative data.

## Conclusion

This study highlighted that stigma can have greater negative consequences that extend beyond just patient–practitioner interactions. Feelings of being judged and/or shunned can lead to patients feeling uncomfortable, unwilling to seek medical attention, or leaving early during an admission without receiving adequate treatment or care. This can lead to negative health consequences being experienced or current health conditions being left untreated and having repercussions for the patient’s future. Enhanced training and education for healthcare providers could help bridge potential divides that may exist between people who use methamphetamine and healthcare providers, but a change of culture is also required for this to be effective. The way people who use methamphetamine are perceived and cared for would also need to be amended in addition to education. Policy changes could also be useful in achieving this goal. Education could help reduce stigma and equip healthcare professionals with the knowledge needed to correctly identify withdrawal symptoms and drug-seeking behavior and provide adequate care.

## Data availability statement

The original contributions presented in the study are included in the article/Supplementary material, further inquiries can be directed to the corresponding author.

## Ethics statement

The study involving humans were approved by Western University Research Ethics Board. The studies were conducted in accordance with the local legislation and institutional requirements. The participants provided their written informed consent to participate in this study.

## Author contributions

CF: Conceptualization, Data curation, Formal analysis, Funding acquisition, Investigation, Methodology, Project administration, Resources, Supervision, Validation, Visualization, Writing – original draft, Writing – review & editing. JS: Data curation, Formal analysis, Investigation, Methodology, Project administration, Resources, Supervision, Validation, Visualization, Writing – original draft, Writing – review & editing. LS: Data curation, Formal analysis, Investigation, Methodology, Project administration, Validation, Visualization, Writing – original draft, Writing – review & editing.
