# Supplementary material for: Perceptions of stigma among people with lived experience of methamphetamine use within the hospital setting: qualitative point-in-time interviews and thematic analyses of experiences
Source: Front Public Health. 2024 Feb 13;12:1279477. doi: 10.3389/fpubh.2024.1279477 (PMC10896942; doi:10.3389/fpubh.2024.1279477)
Supplement: Supplementary file 1 [file Data_Sheet_1.PDF]

Appendices

Appendix A – Interview Guide for People with Lived Experience of Methamphetamine Use

(1) What is your experience with the way things are currently within the hospitals for harm reduction and methamphetamine use?

(2) What are some of the issues with the current approach within the hospitals for harm reduction and methamphetamine use?

(3) What do you think should be changed regarding the current approaches to harm reduction?

(4) What are some aspects you would not change regarding the current approaches to harm reduction?

(5) How should a new approach help you with your goals?

(6) Do you have any other recommendations that may be useful to you or others who use methamphetamine?
